# Associating liver partition and portal vein ligation for staged hepatectomy (ALPPS) as a double-edged sword: leveraging imaging evaluation to maximize benefits and mitigate risks

**DOI:** 10.1186/s13244-026-02247-y

**Published:** 2026-03-17

**Authors:** Mengqi Huang, Chenyu Song, Huasong Cai, Lujie Li, Jiawei Liu, Zhi Dong, Jifei Wang, Yuying Chen, Zhenpeng Peng, Xiaoqi Zhou, Shi-Ting Feng

**Affiliations:** 1 Department of Radiology, Sun Yat-sen University First Affiliated Hospital, Guangzhou, China; 2 Department of Radiology, Tongji Hospital, Tongji Medical College, Huazhong University of Science and Technology, Wuhan, China

**Keywords:** Liver tumor, Associating liver partition and portal vein ligation for staged hepatectomy, Magnetic resonance imaging, Future liver remnant

## Abstract

**Abstract:**

Surgical resection is the primary treatment option for patients with malignant liver tumors. However, not all patients can be treated by liver resection, primarily due to the critical limitation of insufficient remnant liver volume. The Associating Liver Partition and Portal vein Ligation for Staged Hepatectomy (ALPPS) has been explored as a treatment option for patients with insufficient future liver volume, owing to its ability to induce rapid liver growth. Though ALPPS has opened up opportunities for radical resection, it also presents challenges, including high morbidity and mortality. Therefore, a thorough preoperative evaluation of liver function is essential for patients scheduled for ALPPS. Medical imaging plays a crucial role in providing important information for patient selection and safety monitoring during the ALPPS process. This review outlines the current status of ALPPS, detailing its primary imaging evaluation workflow and the recommended modalities based on available clinical evidence. Where applicable, levels of evidence and strength of recommendations are provided to guide imaging-based decision-making throughout the ALPPS process.

**Critical relevance statement:**

This review provides a comprehensive overview of the ALPPS and elucidates the pivotal role of medical imaging throughout the clinical pathway: (1) preoperative patient selection, (2) staged interoperative assessment, and (3) postoperative surveillance, to ultimately improve patient outcomes.

**Key Points:**

- ALPPS is a promising therapy for malignant liver tumors due to its characteristic of increasing the resectability.
- Highly selected patients and effective preoperative and interstage management will improve the outcomes and achieve an acceptable morbidity and mortality.
- Medical imaging offers accurate function and anatomy assessment for ALPPS.

**Graphical Abstract:**

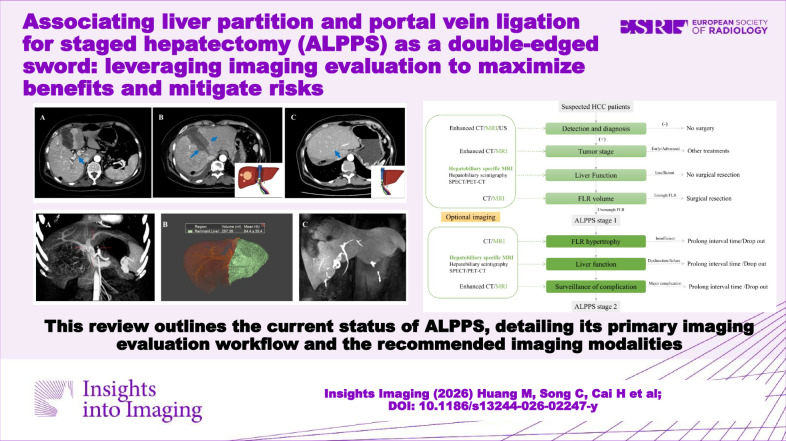

## Introduction

Liver cancer ranks as the sixth most common malignancy and the third leading cause of cancer-related deaths worldwide, with surgical resection remaining the curative treatment for malignant tumors [1–3]. However, surgical intervention is often hindered by excessive tumor size or multifocal hepatic involvement at diagnosis. To increase the resection rate of tumors, surgeons have explored various strategies to address the issue of insufficient future liver remnant (FLR). The commonly accepted threshold for a safe FLR to minimize post-hepatectomy liver failure risk is greater than 20% in patients with normal liver function. This threshold increases to greater than 35% in cases of mildly impaired liver function and to greater than 45% for severely impaired liver function [4]. Clinical strategies include tumor burden reduction via interventional or systemic therapies to indirectly increase the FLR. In addition, FLR can be directly increased by inducing hepatic hyperplasia. This can be achieved through preoperative portal vein embolization (PVE), which promotes slow growth in the contralateral liver lobe, or through the Associating Liver Partition and Portal Vein Ligation for Staged Hepatectomy (ALPPS). The ALPPS technique induces liver regeneration by combining portal vein ligation with liver partitioning, allowing for a second-stage hepatectomy to be performed in a relatively short period [5, 6]. In initially unresectable HCC, ALPPS results in higher resection rates and better medium-term survival compared to transarterial chemoembolization (TACE) alone [7, 8]. Additionally, it achieves resection rates and long-term oncologic outcomes that are comparable to or superior to those of PVE—with or without bridging TACE—despite a higher incidence of perioperative morbidity [7, 9, 10]. Furthermore, ALPPS is considered a more promising therapy due to its ability to facilitate rapid liver regeneration [9].

Over the years, numerous studies have emerged regarding the application of ALPPS for the treatment of various cancers. This novel two-staged surgery, ALPPS, was first introduced through a clinical series from five German centers, enabling the resection of 25 nonresectable primary and metastatic liver cancers by inducing rapid liver growth [11]. Since then, ALPPS has been utilized to treat colorectal cancer with liver metastasis (CRLM), cholangiocarcinoma, HCC, and other liver tumors to achieve radical resection [12–14]. However, most studies on ALPPS show a high risk of complications and surgical mortality. Insufficient future liver remnant volume (FLV)poses a significant risk, particularly as FLV growth may be slow or even not obvious in HCC patients with cirrhosis or CRLM patients with chemotherapy-associated hepatic injury. These patients often have a poor tolerance for complications, even during the less invasive stage 1 procedure [15]. Reportedly, risk adjustment for highly selected patients, the use of less invasive ALPPS techniques, and effective interstage management can reduce morbidity and mortality, potentially matching the standard outcomes of major resections [16]. Medical imaging plays a crucial role in patient selection and interstage management. Among various medical imaging methods, hepatobiliary-specific contrast MRI has shown the best evaluation efficacy by offering accurate assessments of tumor stage, anatomical variations, FLV, liver function, interstage changes, early detection of insufficient liver volume and function growth, and information on complications. This review summarizes the current status of ALPPS and provides a comprehensive overview of medical imaging evaluation throughout the ALPPS procedure.

## Methodological approach and evidence grading

This narrative review focused on the role of medical imaging in patient selection, interstage monitoring, and postoperative surveillance across the ALPPS pathway. We have selectively incorporated high-quality evidence from prospective and retrospective studies, multicenter registries, and clinical guidelines where available.

To enhance clinical applicability and align with current standards in radiology literature, key recommendations regarding imaging modalities and decision-making thresholds are accompanied by an assessment of the Level of Evidence (LoE) and Strength of Recommendation (SoR), based on the evidence grading framework proposed by *Insights into Imaging* [17]. This system categorizes evidence into three levels—High, Moderate, and Low—and links them to the confidence in estimates of benefit/risk and the wording of recommendations (e.g., “should” vs. “may”). Where available, we assign LoE and SoR to imaging-based statements based on study design, validation status, reference standards, and consistency across the literature.

## ALPPS: surgical procedures, clinical benefits, and challenges

**Fig. 1:**
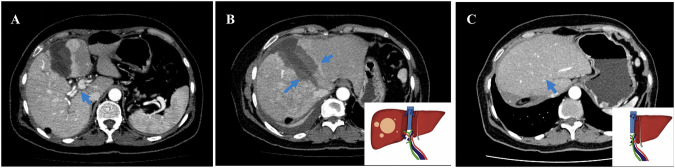
The procedure of ALPPS. **A** Portal vein ligation in the first stage. The maximum intensity projection of the CT image in the portal phase after the ALPPS stage 1 procedure shows no contrast filling in the right portal vein (arrow) as a result of right portal vein ligation. **B** Liver partition in the first stage. The axial enhanced CT image in arterial phase after ALPPS stage 1 procedure shows separation of the liver parenchyma (arrow) between the S4, S5 and S8. **C** Right hepatectomy in the second stage. The axial enhanced CT image after ALPPS stage 2 shows that the right liver lobe and gallbladder have been removed, and enlargement of the left lobe (arrow)

ALPPS is a two-staged hepatectomy (Fig. 1) that involves portal vein ligation or embolization and liver partition in the first stage (Stage 1), followed by final liver resection in the second stage (Stage 2). For patients with extensive liver tumor burden, ALPPS represents a viable surgical option for selected patients with otherwise unresectable hepatic tumors.

## Surgical procedures

### Stage 1: Portal vein ligation or embolization and liver partition

In the first stage, the procedure consists of multiple sequential steps. (i) Abdominal exploration: conventional abdominal exploration is first conducted to exclude the presence of intraperitoneal extrahepatic metastasis [18]. (ii) Portal vein ligation (PVL): Relevant hilar structures, including the common bile duct, portal vein, and hepatic artery, are carefully exposed and identified. The portal vein of the tumor-bearing hemiliver, usually the right portal vein, is then ligated [18]. For patients undergoing further trisegmentectomy, additional portal vein branches of segment IV, with or without segment I, are identified and ligated. (iii) In situ liver partition: the partition line for the subsequent right hepatectomy is determined by temporarily clipping the right hepatic artery and using an anterior approach [19]. The transection line for further right trisegmentectomy is planned near the right side of the falciform ligament, preserving the left and middle hepatic veins, guided by intraoperative ultrasound [11]. A plastic sheet or hemostatic gauze is recommended to be placed on the cover of the injured liver to prevent adhesions [19]. Some experts have raised concerns about the use of plastic materials in the case of insufficient liver regeneration for Stage 2 hepatectomy [20]. Throughout this stage, all hepatic arterial blood flow, venous outflow, and bile ducts are preserved to avoid necrosis, congestion of the liver parenchyma, and bile duct complications [21, 22].

### Stage 2: The final liver resection

In Stage 2, surgeons complete either a right hepatectomy or right trisegmentectomy, which involves dividing the right portal vein, right hepatic artery, right hepatic bile duct, and right hepatic vein, potentially including the middle hepatic vein. Bile leakage is assessed during both Stage 1 and Stage 2 [23]. The interval waiting time between the two stages is determined by the growth rate of FLR and the patient’s overall status.

## Clinical benefits

ALPPS has a key characteristic of rapid liver regeneration that makes it an attractive and popular option for surgeons treating primary or metastatic liver tumors with insufficient FLR. The mechanisms underlying liver growth in ALPPS remain unclear, though several hypotheses have been proposed. From a macro perspective, impaired bilateral portal blood flow significantly enhances the growth of the remnant liver, which is a crucial factor for liver regeneration, along with increased nutrition and hepatotrophic factors [14]. Additionally, the arterialized tumor-bearing liver allows the remnant liver to better tolerate hemodynamic stress and modulate dual hepatic blood flow [24]. The rapid hypertrophy of the FLV in ALPPS is driven by the synergistic interaction of hemodynamic changes and surgical trauma-induced inflammation. Portal vein ligation redirects flow to the FLV, while transection disrupts intrahepatic collaterals—maximizing shear stress and regenerative stimulus, with greater hypertrophy seen in complete versus partial transection [25]. This mechanical stimulus is amplified by the inflammatory response triggered by surgical injury. At the molecular level, Kupffer cell activation leads to the rapid release of IL-6 and TNFα, which initiate pro-regenerative signaling through pSTAT3 and NF-κBp65. Concurrently, the Hippo/YAP pathway is activated, further promoting hepatocyte proliferation [15, 26]. Together, these mechanisms generate a uniquely potent regenerative response.

When compared to other liver hypertrophy methods, such as PVL, portal vein embolization (PVE), or conventional two-stage hepatectomy, ALPPS offers distinct advantages. Most notably, the combination of in situ parenchymal transection and portal occlusion induces rapid FLR hypertrophy, typically achieving a 70–80% volume increase within 7–14 days, in contrast to the 4–6 weeks or longer required after PVE or PVL [23], which will increase the resectability of liver tumors [27]. The markedly shortened interstage interval minimizes the two primary causes of failure to proceed to resection: insufficient FLR hypertrophy and tumor progression during the waiting period. Consequently, while completion hepatectomy rates with PVE-based approaches rarely exceed 60% to 80% [28, 29]. In stark contrast, ALPPS, by inducing rapid liver regeneration within days, dramatically shortens this vulnerable interval, thereby minimizing these risks and achieving a resectability rate that is usually over 90% [6]. This capability also supports the role of ALPPS as a salvage procedure (“rescue ALPPS”) for patients who drop out of PVE or PVL for these reasons [30, 31]. The regeneration of FLR in rescue ALPPS for patients who failed PVE or PVL is comparable to that of other ALPPS series, with median FLR reaching about 88% between the two stages [32].

It should be acknowledged that newer strategies beyond conventional PVE, such as dual venous embolization (DVE; sequential PVE followed by hepatic vein embolization) and liver venous deprivation (LVD), have been developed to accelerate FLR hypertrophy and reduce dropout rates. However, these advanced approaches still require a multi-week waiting time for resection, which contributes to suboptimal completion hepatectomy rates. For instance, in a recent series of 130 patients undergoing PVE or DVE, only 56% proceeded to definitive resection, with tumor progression causing nearly 80% of these failures [33]. Similarly, a multicenter comparison reported successful resection rates of 72.6% for LVD (± rescue ALPPS) versus 90.6% for ALPPS, with ALPPS achieving significantly faster hypertrophy and a significantly shorter interstage interval (median 10 vs. 37 days, *p* < 0.05) [34]. Therefore, while LVD may offer a safer profile in select patients—particularly those with preserved liver function—it does not eliminate the critical risk of disease progression during the waiting period. Given these advantages, ALPPS is emerging as a highly promising strategy for managing primary or secondary liver malignancies, particularly for advanced liver tumors with insufficient FLR [9].

## Challenges

Despite the advantages of faster and more effective liver growth and improved resection rate, ALPPS also carries the risk of increased complications. In the first multicenter study of ALPPS conducted by Schnitzbauer et al, 68% of patients experienced perioperative complications, and there was a 12% in-hospital mortality rate [11]. The study reported complications related to hepatic insufficiency, such as ascites, persistent cholestasis, and sepsis. These complications may be linked to the physiological stress associated with the short interval between the associating liver partition and portal vein ligation for staged hepatectomy operations [35].

Liver cirrhosis or liver injury can further limit liver regeneration and result in even higher morbidity and mortality rates. Post-hepatectomy liver failure (PHLF) remains a leading cause of morbidity and mortality following ALPPS [18]. PHLF is defined by the International Study Group of Liver Surgery (ISGLS) as the presence of an elevated international normalized ratio (INR) and concomitant hyperbilirubinemia occurring on or after postoperative day 5, with severity graded as mild (A), moderate (B), or severe (C) based on the level of clinical intervention required [36]. Importantly, the ISGLS criteria have been validated and applied to assess liver dysfunction after Stage 1 of ALPPS, even in the absence of hepatic resection. For example, the International ALPPS Registry (*n* = 320 patients from 55 centers) reported that 14% of patients met ISGLS criteria for liver failure after Stage 1, a status that strongly predicted subsequent 90-day mortality after Stage 2 [37]. Thus, while Stage 1 does not involve parenchymal resection, ISGLS-based biochemical thresholds provide a clinically validated and prognostically relevant definition of severe interstage liver dysfunction.

**Fig. 2:**
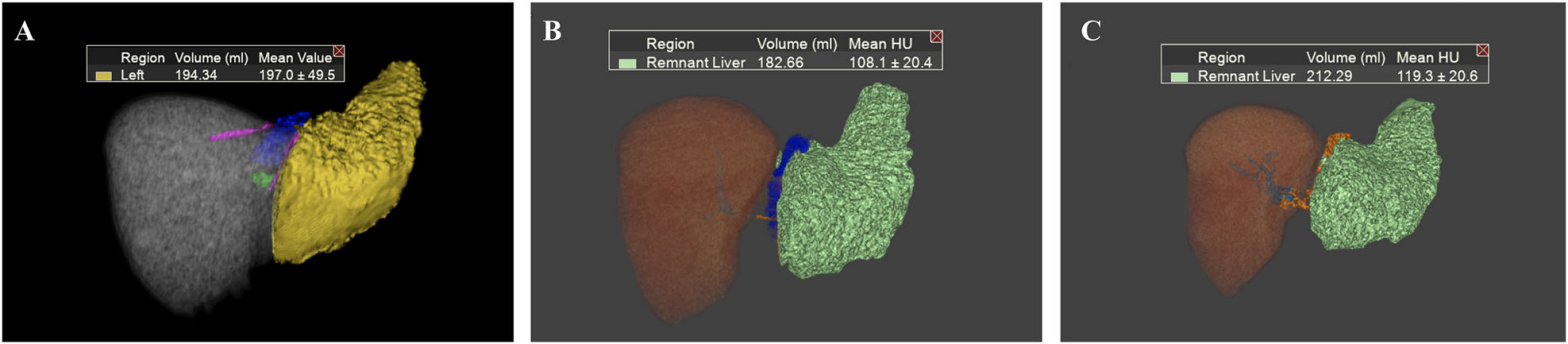
Unsatisfied liver regeneration in the interval stage. **A** 36-year-old male patient, who did not undergo ALPPS stage 2 surgery due to insufficient liver volume. A three-dimensional image of the preoperative MRI shows the liver volume of the left lobe is 194.34 mL. **B** Three-dimensional image of the CT in 7 days after the ALPPS stage 1 shows no significant liver regeneration of the left lobe; the volume is 182.66 mL. **C** Three-dimensional image of the CT in 21 days after the ALPPS stage 1 shows still slow liver regeneration of the left lobe, the volume is 212.29 mL (16.19% of the standard liver volume)

Sufficient regeneration of the FLR during the interval stage of ALPPS is crucial to avoiding postoperative liver failure. Inadequate liver regeneration indicates that the FLR has not met the required threshold (more than 40% for the injured liver) for proceeding to stage 2 (Fig. 2). The severity of fibrosis or cirrhosis was negatively correlated with liver hypertrophy in HCC patients [18]. Other factors that can impair liver regeneration include right portal vein recanalization due to the formation of an arteriovenous fistula between the right portal vein and right hepatic artery, as well as inadequate arterial blood supply to the remnant liver caused by arterial blood diversion to the large HCC lesion [38, 39]. These issues can further compromise the liver’s ability to regenerate effectively.

The indications for ALPPS include an FLV of less than 35% for unilobar lesions, or less than 40% for bilobar lesions, an indocyanine green clearance (ICG) rate of less than 20% at 15 min, Child-Pugh grade A liver function, a serum platelet count greater than 100*10^9^/L, and a patent right portal vein [9]. The contraindications for ALPPS include unresectable lesions, extrahepatic metastasis, portal vein thrombosis, and severe portal hypertension, which may present as ascites and/or varicose veins [9, 18, 40]. In conclusion, although ALPPS has been in use for more than 60 years, its application requires careful patient selection and a focus on refining minimally invasive approaches.

## Imaging assessment across ALPPS surgical phases: Pre-ALPPS, interstage, post-ALPPS

### Pre-ALPPS imaging assessment

**Fig. 3:**
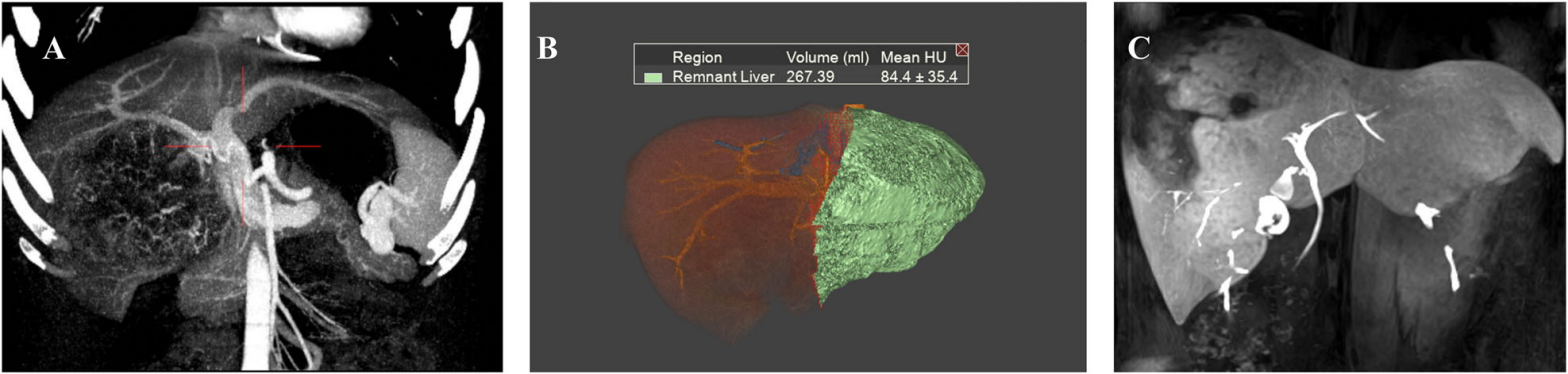
Preoperative assessment with CT and MRI. **A** The maximum intensity projection of a CT image showed the anatomy of the hepatic artery and portal vein, allowing for the identification of the anatomical variations that may interfere with surgical procedures or cause vascular injury. **B** A three-dimensional CT image and virtual right hemi-hepatectomy showed the insufficient liver volume of the left lobe. **C** The maximum intensity projection of an MRI in the hepatobiliary phase showed good liver function and the anatomy of the bile duct: normal contrast enhancement of the hepatic parenchyma, accompanied by normal excretory function of the biliary system

The preoperative assessment before ALPPS Stage 1 primarily focuses on patient selection. This assessment should include not only the basic characteristics of the patient, such as age and anesthesiology risk, but also important factors, including tumor stage evaluation, anatomical variation assessment [15], FLV, and future liver remnant function (FLF)assessment (Fig. 3). Preoperative imaging should be performed within 4–6 weeks before the Stage 1 procedure, particularly for patients with rapidly progressive malignancies such as HCC or colorectal liver metastases [41]. Multiphasic contrast-enhanced CT or MRI—including non-contrast, late arterial, portal venous, and delayed phases (typically at 3 min)—is essential for accurate tumor characterization and vascular mapping [41]. When available, gadoxetate-enhanced MRI with a hepatobiliary phase acquired 20 min post-injection is preferred for its superior lesion detection and functional assessment capabilities [42, 43].

#### Tumor stage and anatomy assessment

Medical imaging, such as computed tomography (CT) and MRI, particularly when using contrast agents, provides high tissue and spatial resolution, enabling precise tumor stage evaluation through accurate lesion detection and characterization. These imaging methods also elucidate the relationship of the tumor with surrounding anatomy (e.g., portal veins, hepatic artery, hepatic veins, bile ducts), and assess the underlying liver disease (e.g., fibrosis, cirrhosis, steatosis, cholestasis), as well as detect anatomical variations [15], which are keys for clinical decision-making.

Anatomic variations of the portal vein, hepatic artery, and bile duct were identified in 11%, 25% and 25% of patients, respectively [44]. The most important anatomical variations relevant to the preoperative evaluation of ALPPS include trifurcation of the portal vein, left portal vein branches supplying segments V and/or VIII, right portal vein supplying segment IV, and shortened lengths of the right portal vein, along with other variants of hepatic artery hepatic venous, and biliary system that can affect hemi-hepatectomy or right thrisegmentectomy [45]. It is noteworthy that variant portal veins are frequently associated with variant bile ducts [44].

#### FLV assessment

The FLV, measured by volumetric CT or MRI, is fundamental to the preoperative assessment, as it is an important factor in determining whether major hepatectomy or ALPPS is appropriate for the patient. The most commonly used and recommended calculation method currently of FLR is based on the standardized total liver volume (sTLV), calculated as follows:

$${{{\rm{sFLR}}}}( \% )=({{{\rm{FLV}}}}/{{{\rm{sTLV}}}})\times 100$$

FLV: Future liver volume (mL), derived from semi-automated segmentation software on preoperative imaging. sTLV: Standardized total liver volume (mL), estimated based on body surface area (BSA) using the widely validated formula by Vauthey et al [46].

$${{{\rm{sTLV}}}}\left({{{\rm{mL}}}}\right)=706.2\times {{{\rm{BSA}}}}({{{{\rm{m}}}}}^{2})+2.4$$

The BSA was calculated using the Du Bois equation: BSA (m²) = 0.007184 × Height (cm)^0.725^ × Weight (kg)^0.425^.

Studies have shown that better outcomes can be achieved in HCC patients with an FLR of more than 30% of the estimated standard liver volume, Child-Pugh A cirrhosis, and good liver function, such as an indocyanine green retention rate of less than 20% at 15 min [19]. Conversely, more than 40% of FLR is needed for HCC patients with underlying liver injury to proceed with major hepatectomy [11]. These thresholds are associated with significantly lower morbidity and mortality and are supported by multiple observational studies and expert consensus (LoE: Moderate; SoR: Should be used for patient selection).

Both CT and MRI provide high reproducibility in liver volumetry. CT remains the most widely used modality due to its broad availability, short acquisition time, and excellent spatial resolution [47] (LoE: Moderate; SoR: Should be considered as a standard initial modality). However, volumetric MRI demonstrates comparable accuracy and repeatability to CT [48], while offering additional advantages such as absence of ionizing radiation, superior soft-tissue contrast, and improved detection of small satellite lesions during the hepatobiliary phase [3]. For centers with expertise, gadoxetate-enhanced MRI is increasingly considered the preferred modality for comprehensive preoperative assessment, especially in patients with chronic liver disease (LoE: Moderate; SoR: May be preferred in centers with expertise, particularly for patients with chronic liver disease).

#### FLF assessment

Preoperative assessment of liver function and regional liver function is important for patients scheduled for ALPPS. Traditional global tests—including Child-Pugh score, MELD, and indocyanine green clearance (ICG-R15)—assess overall liver function but may overestimate functional reserve in the context of segmental portal deprivation, making them suboptimal for FLR-specific decision-making [49].

Advanced imaging techniques allow for regional functional assessment, providing more accurate insights into the metabolic capacity of the remnant liver. Hepatobiliary-specific contrast-enhanced MRI (e.g., gadoxetate) enables quantitative assessment of hepatocyte uptake in the hepatobiliary phase [50]. This method correlates well with postoperative liver function and has been prospectively validated in several cohorts (LoE: Moderate, SoR: Should be considered for FLFevaluation in ALPPS candidates). 99 mTc-GSA SPECT/CT measures asialoglycoprotein receptor density, strongly correlating with hepatocellular function and PHLF risk [51, 52] (LoE: Moderate; SoR: May be considered for functional assessment of the future liver remnant in selected centers with nuclear medicine expertise). 2-[¹⁸F]fluoro-2-deoxy-D-galactose (FDGal) is a galactose analog selectively taken up by hepatocytes via the asialoglycoprotein receptor and metabolized intracellularly, enabling quantitative, regional assessment of metabolic liver function. In a prospective study of patients with cirrhosis, FDGal-PET/CT demonstrated heterogeneous intrahepatic functional distribution and provided reproducible kinetic parameters (e.g., K_met) that reflect regional metabolic capacity [53] (LoE: Low; SoR: Could be considered in specialized centers for functional mapping when MRI is inconclusive, particularly in cirrhotic patients).

While nuclear medicine techniques require specialized infrastructure and longer processing times, hepatobiliary-specific contrast-enhanced MRI provides a practical, radiation-free method for simultaneous assessment of FLV and FLF [54]. It can be readily integrated into routine preoperative imaging protocols and has been associated with improved risk stratification for post-hepatectomy liver failure (PHLF) in patients undergoing major hepatectomy [55] (LoE: Moderate; SoR: Should be considered as part of the standard preoperative imaging workup in centers performing ALPPS or extended liver resections).

A summary of the key characteristics and evidence base for these functional imaging modalities is provided in Table 1.

**Table 1:** Comparative analysis of imaging modalities for functional assessment

Modality	Mechanism	Availability	SoR [17]	Advantages	Limitations
Gadoxetate-enhanced MRI	Hepatocyte uptake (OATP1B1/B3)	High (in hepatobiliary MRI centers)	Strong	No radiation; high spatial resolution; combined volumetric and functional assessment; suitable for serial monitoring	Limited in cholestasis; requires breath-hold; variable uptake in severe liver disease
^99m^Tc-GSA SPECT/CT	ASGPR receptor binding	Limited (requires nuclear medicine expertise)	Conditional	Validated correlation with hepatocellular function and PHLF risk; useful in cirrhotic patients	Limited availability; radiation exposure; longer acquisition time
^99m^Tc-mebrofenin scintigraphy	Organic anion transport and biliary excretion	Limited	Conditional	Dynamic functional data; established use in liver surgery	Lower spatial resolution; less sensitive to regional metabolic changes
FDGal-PET/CT	Metabolic trapping via ASGPR and hexokinase	Very limited (research only)	Conditional	Quantitative kinetic modeling of galactose metabolism; high sensitivity to functional heterogeneity	Not clinically available; requires cyclotron; expensive; minimal data in ALPPS
ICG-R15	Global liver function	Universal	Conditional	Widely accessible; simple to perform	

Strength of recommendation: Strong: The modality is recommended for most patients undergoing ALPPS or extended hepatectomy due to a favorable balance of benefits, accuracy, and safety. Conditional: The choice depends on institutional expertise, availability, and patient-specific factors

*ALPPS* associating liver partition and portal vein ligation for staged hepatectomy, *ASGPR* asialoglycoprotein receptor, *PHLF* post-hepatectomy liver failure, *PVL/PVE* portal vein ligation/embolization, *SOR* strength of recommendation

### Interstage imaging assessment

The first interstage imaging assessment for patients without complications is typically performed about 1 week after Stage 1, when FLR regeneration is expected to be achieved. If the hypertrophy is insufficient, repeat imaging is conducted weekly for up to 4 weeks [6].

#### Hypertrophy assessment of the FLR

**Fig. 4:**
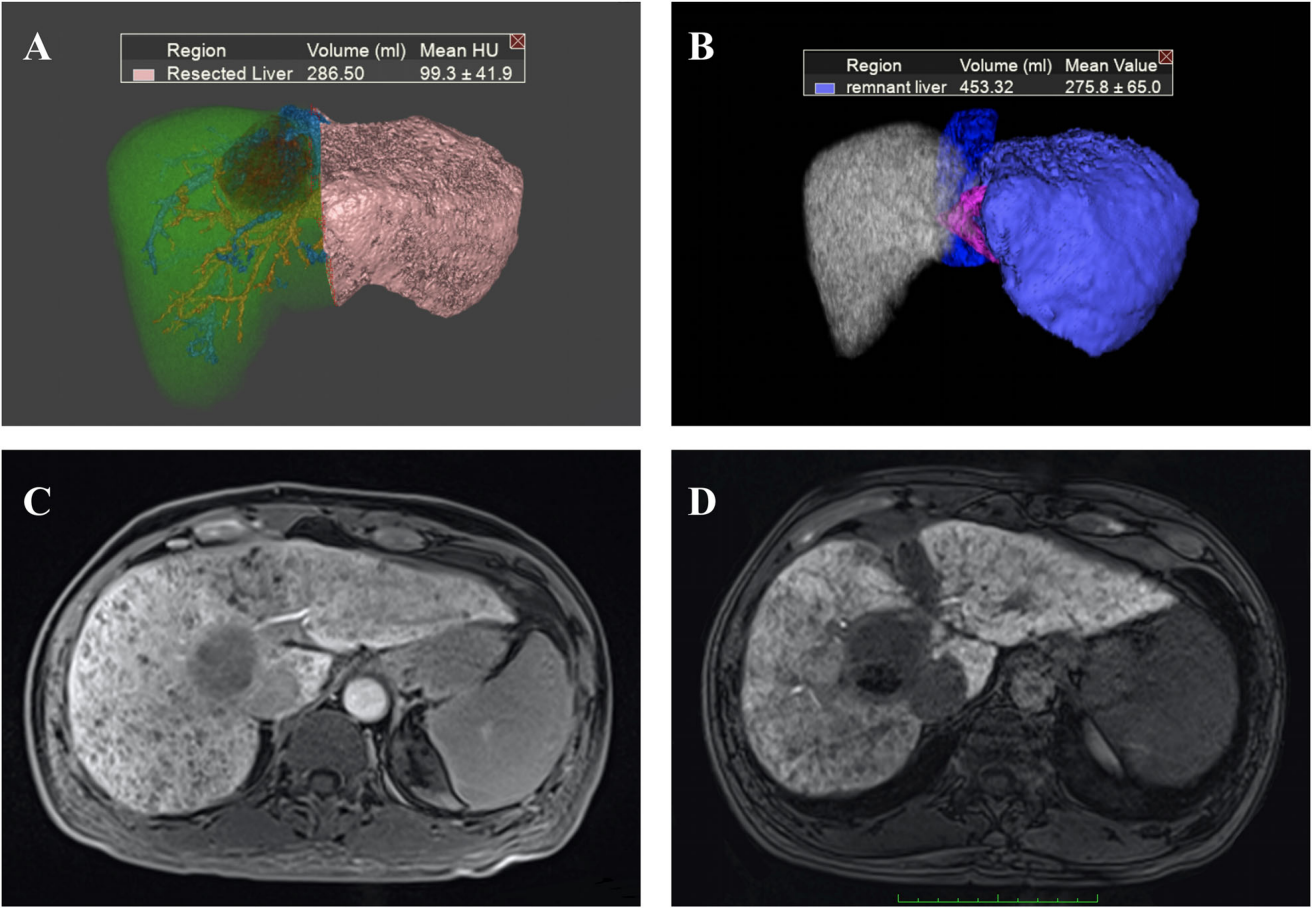
Interstage liver growth and liver function assessment. **A** A 48-year-old male patient, who underwent ALPPS stage 2 surgery after the MRI evaluation of the left lobe volume and function. Preoperative three-dimensional CT and virtual right hemi-hepatectomy show the liver volume of the left lobe is 286.50 mL (22.66% of the standard liver volume). **B** Three-dimensional MRI in 78 days after ALPPS stage 1 shows the liver volume of the left lobe is 453.32 mL (35.84% of the standard liver volume). **C** Axial MRI in hepatobiliary phase in 9 days after ALPPS stage 1 shows liver cirrhosis and dysfunction of the liver parenchyma: heterogeneous contrast enhancement with moderate intensity. **D** Axial MRI in hepatobiliary phase in 78 days after ALPPS stage 1 shows improved liver function, particularly in the left lobe: persistent heterogeneous parenchymal contrast uptake, but with significantly intensified enhancement (and increased volume as demonstrated on **B**), the patient proceeds to stage 2 soon after the examination

The main goal of the first post-Stage 1 imaging examination is to assess the growth of the FLR (Fig. 4) and to identify early factors that may affect its development. The widely accepted criteria for proceeding to Stage 2 include an FLR greater than 30% of the standard liver volume (SLV) in patients with a healthy liver or an FLR greater than 40% SLV in those with underlying liver injury (e.g., cirrhosis, chemotherapy-induced steatohepatitis) [56]. These thresholds are associated with significantly lower rates of post-hepatectomy liver failure and are supported by multiple observational studies (LoE: Moderate, SoR: These volumetric thresholds should be used to guide the timing of Stage 2 surgery in clinical practice). However, volumetric evaluation of the FLR alone may not accurately reflect its functional reserve [57]. The increase in liver volume may not be accompanied by a similar degree of functional growth, leading to an overestimation of the functional reserve and potentially affecting the timing of Stage 2 [58, 59] (Level of Evidence: Moderate; Recommendation: Volumetric criteria alone should not be used to determine Stage 2 timing without concurrent functional assessment). This discrepancy can be explained by findings from Matsuo et al, which indicate that in areas of rapidly regenerated FLR in ALPPS patients, there tends to be a higher presence of immature cells, characterized by bright (glycogen-rich), dense, small hepatocyte cells and narrowed sinusoids, compared to the slowly hypertrophied FLR in PVE patients [60] (Level of Evidence: Low; Recommendation: These findings underscore the biological plausibility of functional–volumetric discordance and support the integration of functional imaging into interstage evaluation). Consequently, functional assessment of the remnant liver is as critical as volumetric measurement during the interstage period to ensure safe surgical timing [59].

#### Functional assessment of the remnant liver

Currently, there are various methods to assess FLF, including the Child-Pugh score, MELD score, indocyanine green test, technetium-99m galactosyl human serum albumin(99mTc-GSA) SPECT/CT, 99mTc-mebrofenin, 2-[¹⁸F]fluoro-2-deoxy-D-galactose PET/CT, and the hepatobiliary-specific contrast-enhanced MRI [53, 61, 62]. The FLF is a critical determinant of post-Stage 2 mortality in ALPPS [63]. However, not all methods are suitable for assessing the interstage liver function reserve in ALPPS patients. For example, the Child-Pugh score and MELD score are general evaluation tools that do not accurately reflect the FLF (LoE: Low; SoR: Should not be used to assess FLF in ALPPS). The indocyanine green test is also not appropriate for assessing functional changes during FLV hypertrophy in the interstage period, as an increase in liver function in the remnant liver and a decrease in liver function in the deportalized lobe may result in no net effect on total liver function. Additionally, this test depends on the overall blood flow of the liver, which can be changed due to portal vein ligation or embolization [15] (LoE: Low; SoR: Not recommended for guiding Stage 2 timing in ALPPS). In contrast, imaging-based regional functional modalities enable direct quantification of the remnant liver metabolic capacity alongside volumetric hypertrophy [54, 55] (Fig. 4). The FLF can be estimated as the ratio of tracer uptake or hepatic extraction in the remnant liver to that of the total liver—expressed as a percentage—using techniques such as ⁹⁹ᵐTc-GSA SPECT/CT or gadoxetate-enhanced MRI [54, 63]. ⁹⁹ᵐTc-GSA SPECT/CT and ⁹⁹ᵐTc-mebrofenin scintigraphy provide validated, quantitative measures of regional hepatocellular receptor density and excretory function, with demonstrated correlation to post-hepatectomy outcomes in major liver resection cohorts (LoE: Moderate; SoR: May be used for remnant liver functional assessment in centers with nuclear medicine expertise). FDGal-PET/CT enables kinetic modeling of regional galactose metabolism and has shown feasibility in cirrhotic patients, though data in ALPPS remain limited [53] (LoE: Low; SoR: Could be considered in research settings or when other modalities are unavailable). Gadoxetate-enhanced MRI allows simultaneous volumetric and functional evaluation without ionizing radiation, with reproducible quantitative parameters (e.g., hepatobiliary phase uptake, T1 relaxation mapping) linked to PHLF risk (LoE: Moderate; Recommendation: Should be considered the preferred method for integrated FLR assessment in ALPPS when available). Thus, regional functional imaging—not global scores—should guide interstage management to avoid underestimating functional immaturity despite apparent volumetric growth. However, despite advances in the functional assessment of the remnant liver, current research is limited by moderate study sizes and a lack of established threshold criteria, warranting further investigation.

#### Limiting factors of the regeneration of the remnant liver

Although ALPPS has been reported as a potentially promising treatment for malignant liver tumors. The failure rate is approximately 5%, with over 50% of patients experiencing delays in progressing to ALPPS Stage 2, primarily due to insufficient growth of the FLR [64]. Several factors can impact the growth rate of the FLR and prolong the interstage period, potentially leading to dropout from the ALPPS Stage-2 procedure.

**Fig. 5:**
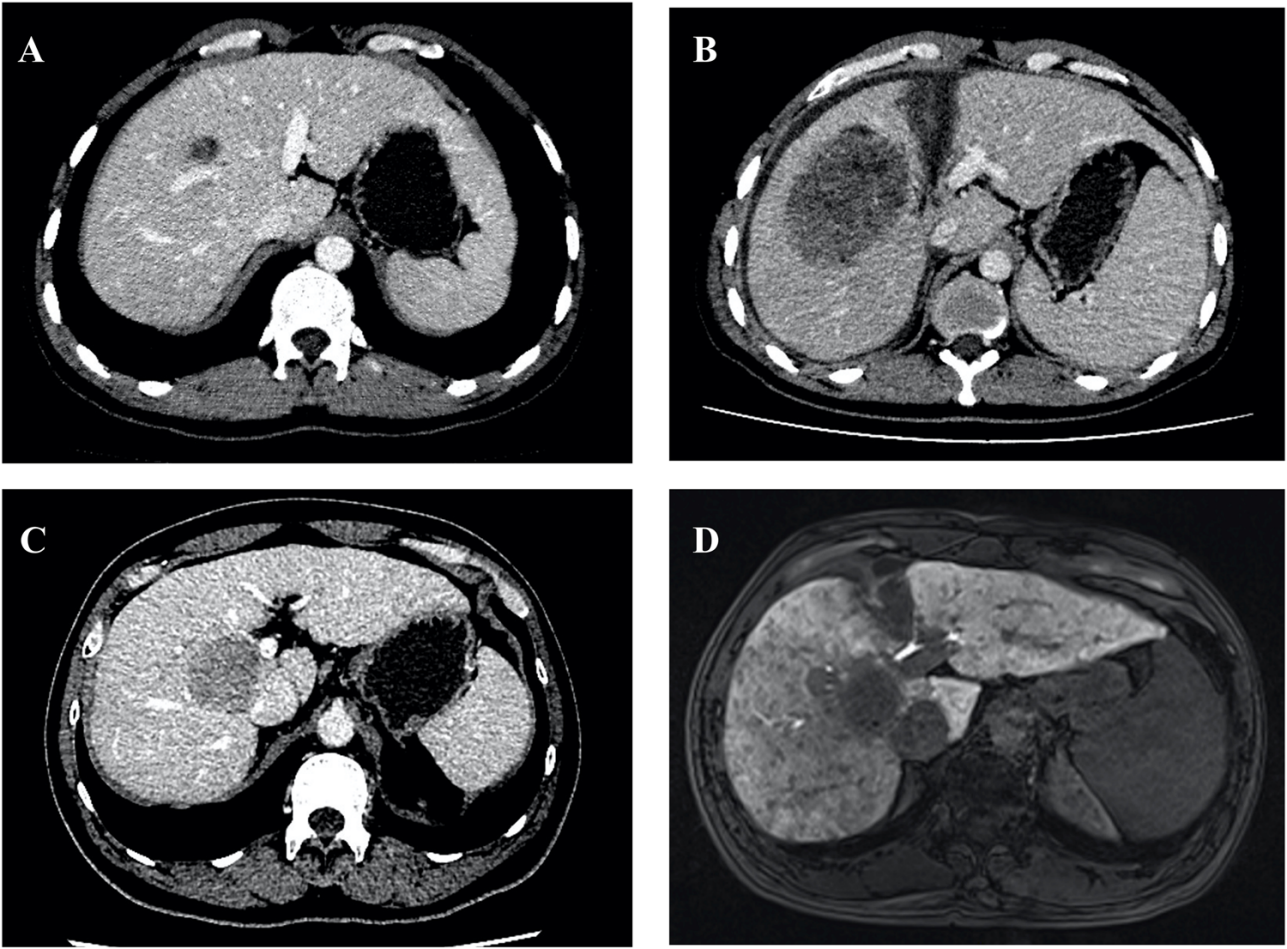
Liver regeneration negatively correlated with fibrosis/cirrhosis. **A** A 32-year-old male patient with mild fibrosis and his preoperative CT image. **B** The same patient in **A** with mild fibrosis. The CT taken 7 days after ALPPS Stage 1 showed that the volume of the left lobe is 49.47% of the standard liver volume, with a kinetic growth rate (KGR) of 46.84 mL/d. **C** A 48-year-old male patient with cirrhosis and his preoperative CT image. **D** The same patient in **C** with cirrhosis. The hepatobiliary phase of MRI in 78 days after ALPPS Stage 1 showed that the volume of the left lobe is 35.84% of the standard liver volume, with a kinetic growth rate (KGR) of 7.61 mL/d, which is significantly slower than that of the patient with mild fibrosis

The severity of liver fibrosis and cirrhosis is a major limiting factor for liver hypertrophy, particularly in HCC patients with hepatic B or C virus infections (Fig. 5). It has been demonstrated that the kinetic growth rate of the remnant liver is negatively correlated with the severity of fibrosis and cirrhosis [65, 66]. Most cases of HCCs are associated with chronic HBV or HCV infections [67], which often progress into fibrosis or cirrhosis. In such cases, excessive accumulation of the extracellular matrix and degeneration of tissue function hinder liver regeneration [68]. Therefore, preoperative assessment of fibrosis may enhance patient selection for ALPPS.

**Fig. 6:**
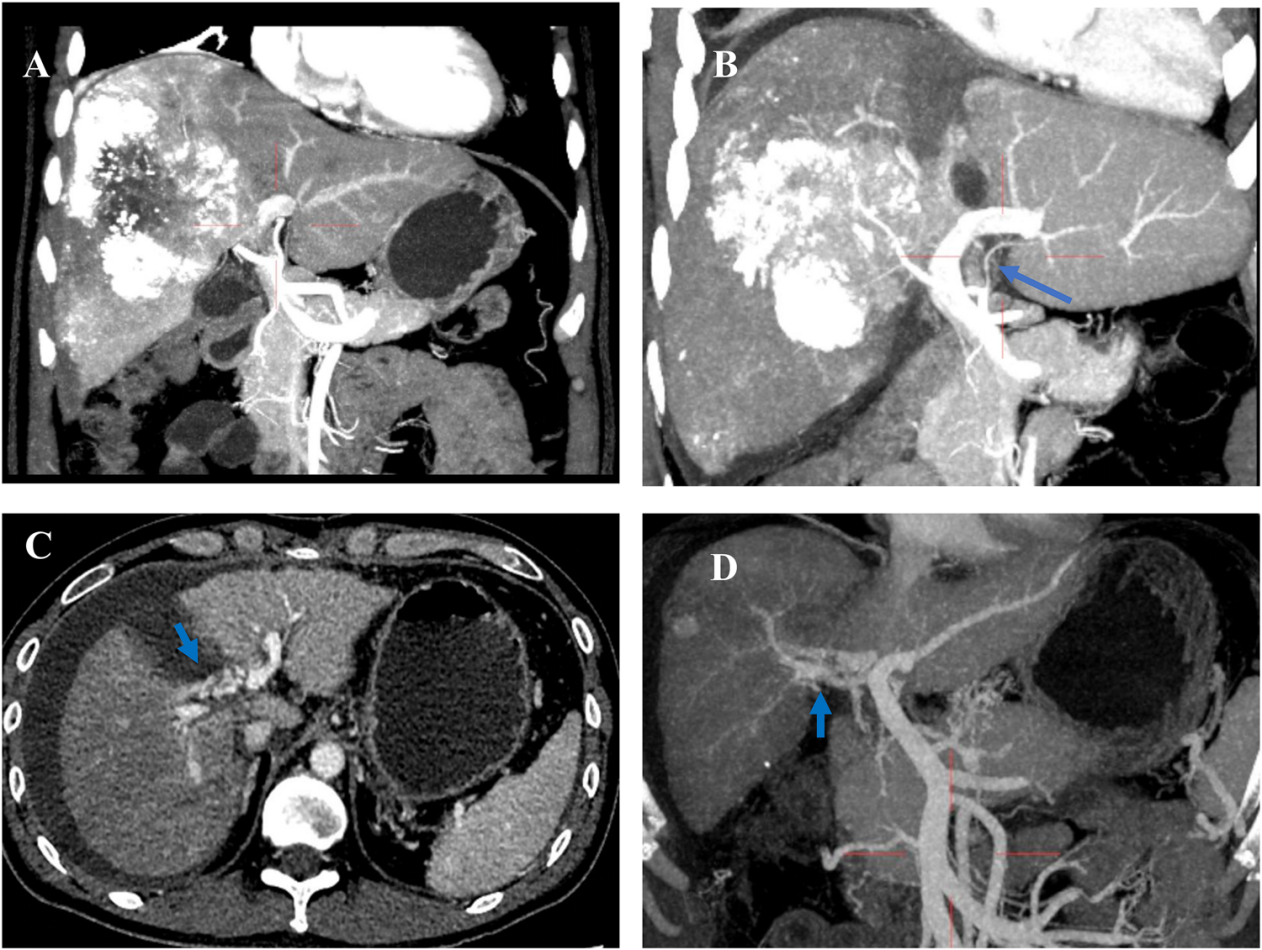
Inadequate liver regeneration due to hemodynamic changes. **A** A 65-year-old male patient, the maximum intensity projection of the preoperative CT image in the arterial phase clearly shows the hepatic artery; this patient underwent TACE treatment before. **B** The same patient in **A**; the maximum intensity projection of the arterial phase CT image 7 days after ALPPS stage 1 shows a smaller left hepatic artery branch (arrow) due to the “arterial steal ” by the huge HCC lesion with inadequate liver regeneration of the left lobe. **C** A 36-year-old male patient, the axial CT in portal phase in 7 days after ALPPS stage 1 shows recanalization of the right portal vein due to the arteriovenous fistula (arrow) between the right hepatic artery with right portal vein. **D** The same patient in **C**; the maximum intensity projection of the portal phase CT image 7 days after ALPPS stage 1 identifies the recanalization(arrow) of the right portal vein

Additionally, hemodynamic redistribution in the portal and arterial blood flow of the diseased liver plays a pivotal role in inducing rapid regeneration of the remnant liver [69]. Hemodynamic changes can lead to inadequate liver regeneration (Fig. 6). For instance, insufficient arterial blood supply to the remnant liver may occur due to diversion of arterial blood away from the remnant liver by a large hepatic tumor or recanalization of the right portal vein due to an arteriovenous fistula between the right hepatic artery and right portal vein [39].

#### Normal findings in CT or MRI

**Fig. 7:**
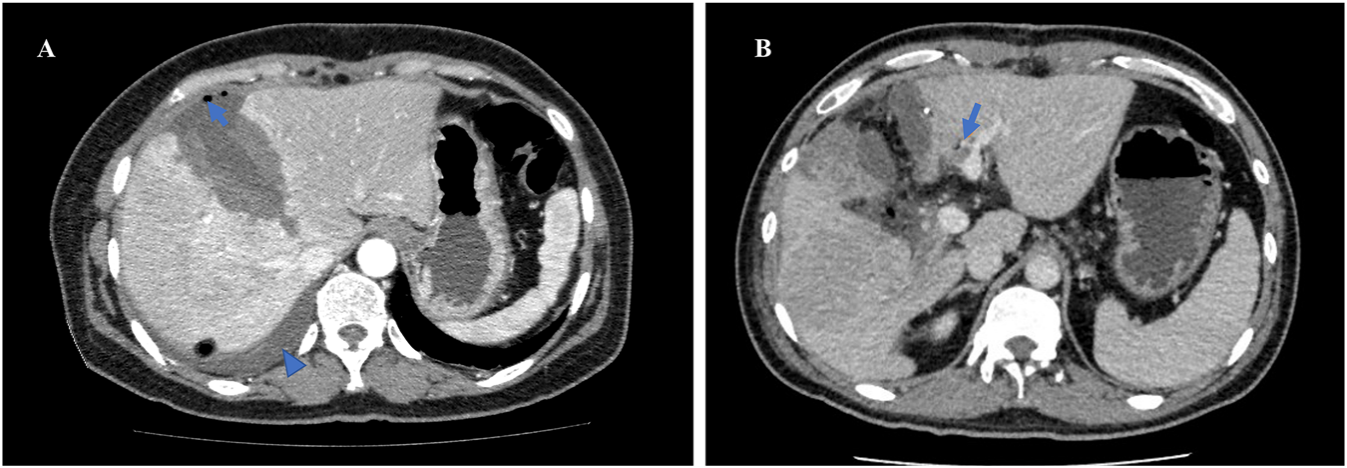
Normal findings and complications after stage 1. **A** A 57-year-old female patient, axial enhanced CT after ALPPS Stage 1 showed normal postoperative findings, including heterogeneous hyperenhancement of the liver parenchyma in the right lobe and at the cut edge, as well as a small amount of gas (arrow) and liquid (arrowhead) in the surgical area and right pleural cavity. **B** A 38-year-old male patient, axial enhanced CT after ALPPS Stage 1 in another patient, showed abnormal thrombosis in the left internal portal vein branch (arrow)

Hypertrophy of the FLV and interrupted blood flow in the right portal vein are normal findings observed in interstage enhanced CT or MRI examination. Additionally, one may see heterogeneous hyperenhancement of the liver parenchyma during the arterial phase, along with a thin hypoattenuation halo surrounding the intrahepatic portal veins in the portal phase. The right branch of the hepatic artery may appear slightly larger than in preoperative images. In addition, the division of liver parenchyma at the resected plane and associated accumulation of small amounts of gas and fluid can also be noted (Fig. 7). On hepatobiliary phase MRI, normal remnant liver parenchyma demonstrates homogeneous and markedly high intensity. In contrast, within the deportalized liver, low-intensity lesions are present, and its parenchyma typically demonstrates relatively reduced intensity on the hepatobiliary phase MRI, suggesting regional functional impairment in the deportalized liver. It is crucial to distinguish these normal findings from signs of infection, which are typically accompanied by symptoms and may present as larger, lenticular or round lesions that are sometimes well-encapsulated on enhanced CT or MRI [40].

#### Interstage complications

After Stage 1, about 58% of patients will experience one or more complications, with about 26% of patients suffering from major complications (classified as > IIIb according to the Clavien-Dindo classification) [70, 71]. Reported interstage complications include bile leakage, fluid collections, hemorrhage, cholangitis, thrombosis of the portal vein, hepatic vein, or hepatic artery, hepatic dysfunction or liver failure, persistent ascites, pleural effusion, prolonged ileus, coagulation disorders, and dysfunction of the cardiovascular, respiratory, or renal systems, as well as encephalopathy and infection [70, 72, 73].

**Fig. 8:**
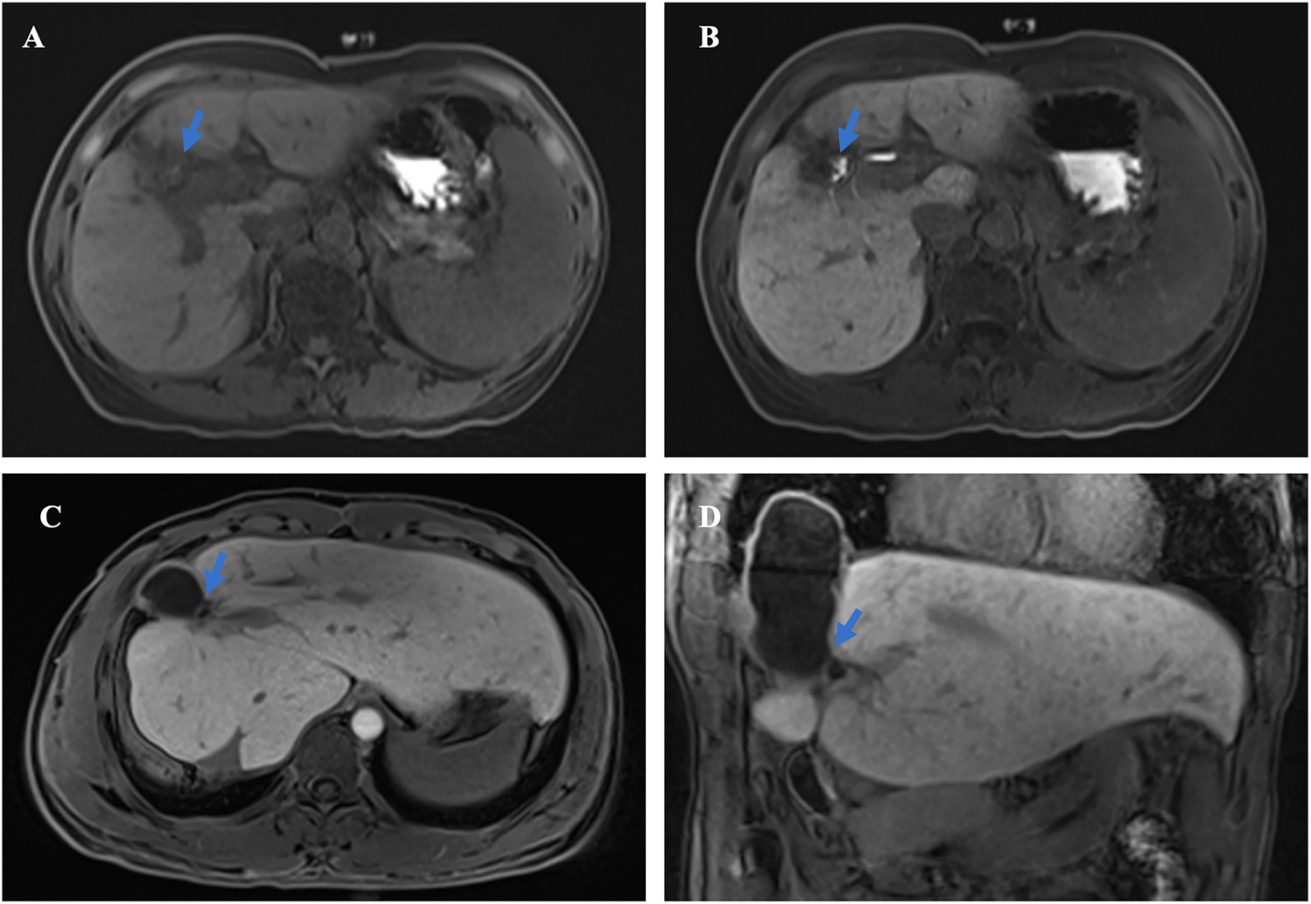
Bile leakage and bile lake. **A** A 49-year-old female patient with bile leakage after partial hepatectomy. Axial pre-contrast phase of MRI shows no significant hyperintensity. **B** The same patient in **A**. Axial hepatobiliary phase of MRI shows the active bile leakage with hyperintensity. **C** A 28-year-old male patient with continuous cutaneous bile leakage after right hemi-hepatectomy. The axial hepatobiliary phase of MRI shows the bile lake near the left bile duct with no contrast filling in and the connection between the cyst and the left bile duct (arrow). **D** The same patient in **C**. The coronal hepatobiliary phase of MRI shows the connection between the cyst and the left bile duct (arrow)

Bile leakage is a common complication after Stage 1, and precise localization of the bile leakage is crucial for determining the best treatment approach. Various techniques are available for accurately identifying bile leakage, including ultrasound, CT and MRI, with MRI being the most effective method for clearly depicting “active” bile leakage due to the excretion of heapability-specific contrast agent from the bile duct [74]. While direct contrast extravasation signifies active leakage, the contrast agent often does not go into the bile lake due to the high internal pressure (Fig. 8). The most common location of leakage is the transection surface of the deportalized liver due to ischemia and parenchymal splitting, and this complication occurs frequently in ALPPS procedures involving trisegmentectomy.

Medical imaging is recommended for symptomatic patients to evaluate the position and severity of the fluid collections, hemorrhage, and to rule out related vascular, biliary, or parenchymal causes (Fig. 7). Among these complications, fluid collections, bleeding, and vascular thrombosis are the most commonly observed interstage issues on CT or MRI. Furthermore, enhanced CT or MRI can provide valuable information about the location of vascular injuries in case of active bleeding [73].

It is noteworthy that liver failure after Stage 1 is a major contributor to 90-day mortality, with 75% of these patients dying from postoperative liver failure and 40% developing liver failure after Stage 1 [21, 37, 75]. Interstage complications, particularly liver dysfunction or failure, necessitate consideration of postponing or delaying the Stage 2 procedure until the liver has recovered [69]. Regional liver function assessment tests can provide direct visualization of liver dysfunction or failure, offering important information about changes in liver function during the interstage period and assisting in decision-making.

### Postoperative assessment and follow-up

**Fig. 9:**
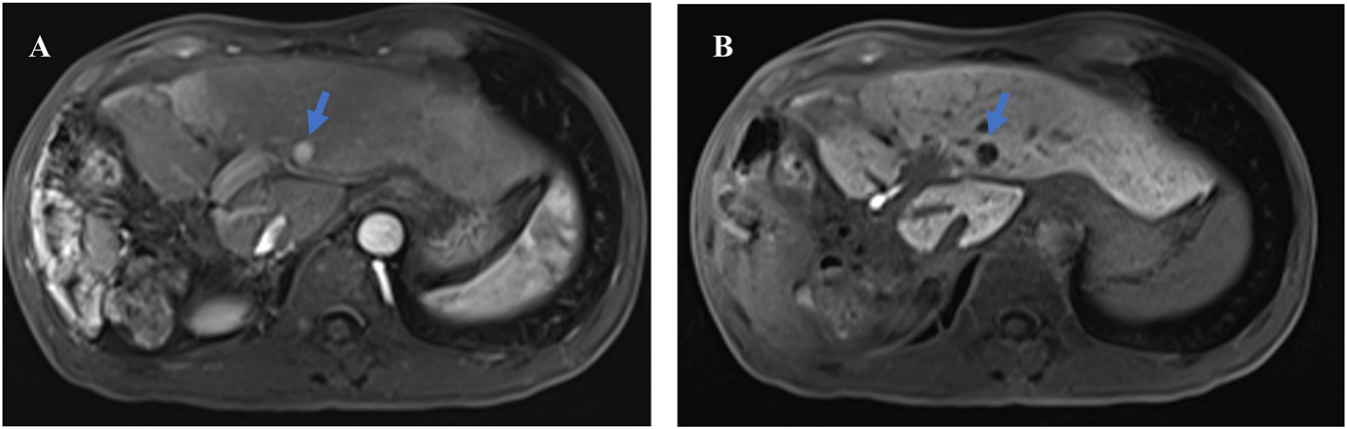
Follow up with tumor recurrence. **A** A 55-year-old male patient, axial follow-up MRI taken 3 months after ALPPS Stage 2 showed an obvious hyperenhancement nodule (arrow) in the arterial phase: suggestive of recurrence. **B** The same 55-year-old male patient, axial follow-up MRI taken 3 months after ALPPS Stage 2 showed an obvious hypointensity nodule (arrow) in the hepatobiliary phase, indicating intrahepatic recurrence that requires early local therapy

The main goals of postoperative assessment after Stage 2 include the identification of potential complications and ongoing follow-up. CT or MRI is recommended for symptomatic patients after ALPPS stage 2 to identify any suspicious complications. Dynamic enhanced CT or MRI is recommended every 3–6 months during the first 2 years postoperatively for early recurrence detection, aligning with HCC surveillance guidelines (Fig. 9) [76]. Although this surveillance strategy is widely adopted in clinical practice, its optimal timing and yield specifically in patients undergoing ALPPS have not been prospectively validated (Level of Evidence: Low; Recommendation: This surveillance interval may be applied to ALPPS patients, but individualized adjustments based on tumor biology, liver function, and institutional experience are reasonable).

#### Normal findings in CT or MRI

Familiarity with normal postoperative image findings is essential for the early detection of postoperative complications. In CT or MRI conducted a few weeks after Stage 2, small amounts of air or fluid collection are normal findings in asymptomatic patients. Air is usually absorbed more quickly than fluid, which may persist for up to 2 months after surgery [77]. The appearance of a hypoattenuating, hypo- or non-enhancing band near the resecting edge of the liver is also frequently seen in about 30–50% of cases after liver resection [77]. Similar to its interstage imaging characteristics, normally functioning liver parenchyma presents with rapid and homogeneous enhancement during the arterial and portal vein phases, uniform hyperintensity in the hepatobiliary phase, and timely contrast excretion into the biliary tree in the excretory phase. In addition, transient splenomegaly accompanied by liver enlargement within 6 months after surgery is a common finding in CT or MRI [40, 78].

#### Postoperative complications

**Fig. 10:**
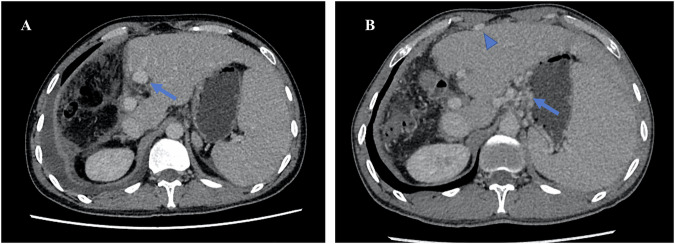
Progressed portal vein hypertension. **A** A 33-year-old male patient, axial CT taken 7 days after ALPPS Stage 2 showed a widened left portal vein (arrow) with no obvious esophageal or gastric varices. **B** The same patient in **A**, axial CT taken 6 months after ALPPS Stage 2 showed newly developed esophageal and gastric varices (arrow), as well as umbilical vein (arrowhead), and splenomegaly. The spleen showed mild restoration at 18 months after Stage 2

Postoperative complications after Stage 2 can be categorized into early complications and late complications [77] based on the time interval from surgery. Early complications usually occur within a few weeks after stage 2 and may include fluid collections, hemorrhage, vascular thrombosis, and hepatic dysfunction or liver failure. In the early stage after surgery, hepatobiliary-specific contrast-enhanced MRI can be used to evaluate liver growth and the function of the remnant liver in case of liver dysfunction or failure. Late complications generally occur more than 3 months after Stage 2, with the most common being biliary complications, such as biliary duct stricture and fistula. MRCP and hepatobiliary-specific contrast-enhanced MRI are suitable to detect the location of biliary duct strictures and biliary fistulas [79]. Additionally, portal hypertension may progress (Fig. 10) due to underlying liver fibrosis or cirrhosis in the remnant liver and increased portal supply. The gold standard for diagnosing portal hypertension is the hepatic venous pressure gradient (HVPG) measurement, which directly reflects the true portal pressure. However, this method is invasive, requiring catheter insertion via the jugular or femoral vein into the hepatic vein. Imaging modalities serve as the primary non-invasive method for diagnosing portal hypertension and assessing its severity. Although they do not directly measure pressure values, they enable the diagnosis of portal hypertension through the observation of a series of indirect signs. CT and MRI (particularly contrast-enhanced scans and angiography) provide comprehensive and detailed visualization of the portal venous system anatomy. They clearly demonstrate dilation of the main portal vein and its branches, allowing non-invasive portography, and three-dimensionally display portosystemic collateral vessels—such as esophageal and gastric varices, splenorenal shunts, and recanalization of the umbilical vein, which constitute direct imaging evidence of portal hypertension. Additional diagnostic imaging features include measurement of portal vein diameter (a diameter > 13 mm in the main portal vein is considered abnormal), as well as evaluation of liver morphology, splenomegaly, and ascites [80].

### ALPPS: developments

Investigators have been working to refine surgical techniques for ALPPS to minimize invasiveness, avoid adhesion, reduce bleeding, and mitigate the risk of bile leakage, all with the goal of decreasing morbidity and mortality rates. These techniques include laparoscopic approaches, modified anterior approaches, robotic-assisted approaches, microwave or radiofrequency ablation-assisted ALPPS, partial ALPPS or mini ALPPS, associating liver tourniquet and partial ligation for staged hepatectomy, and ALPPS combined with portal vein embolization [24, 81–85]. However, it remains uncertain which modified technique can achieve the same liver growth effects as traditional ALPPS while effectively reducing surgical risks.

## Conclusions

**Fig. 11:**
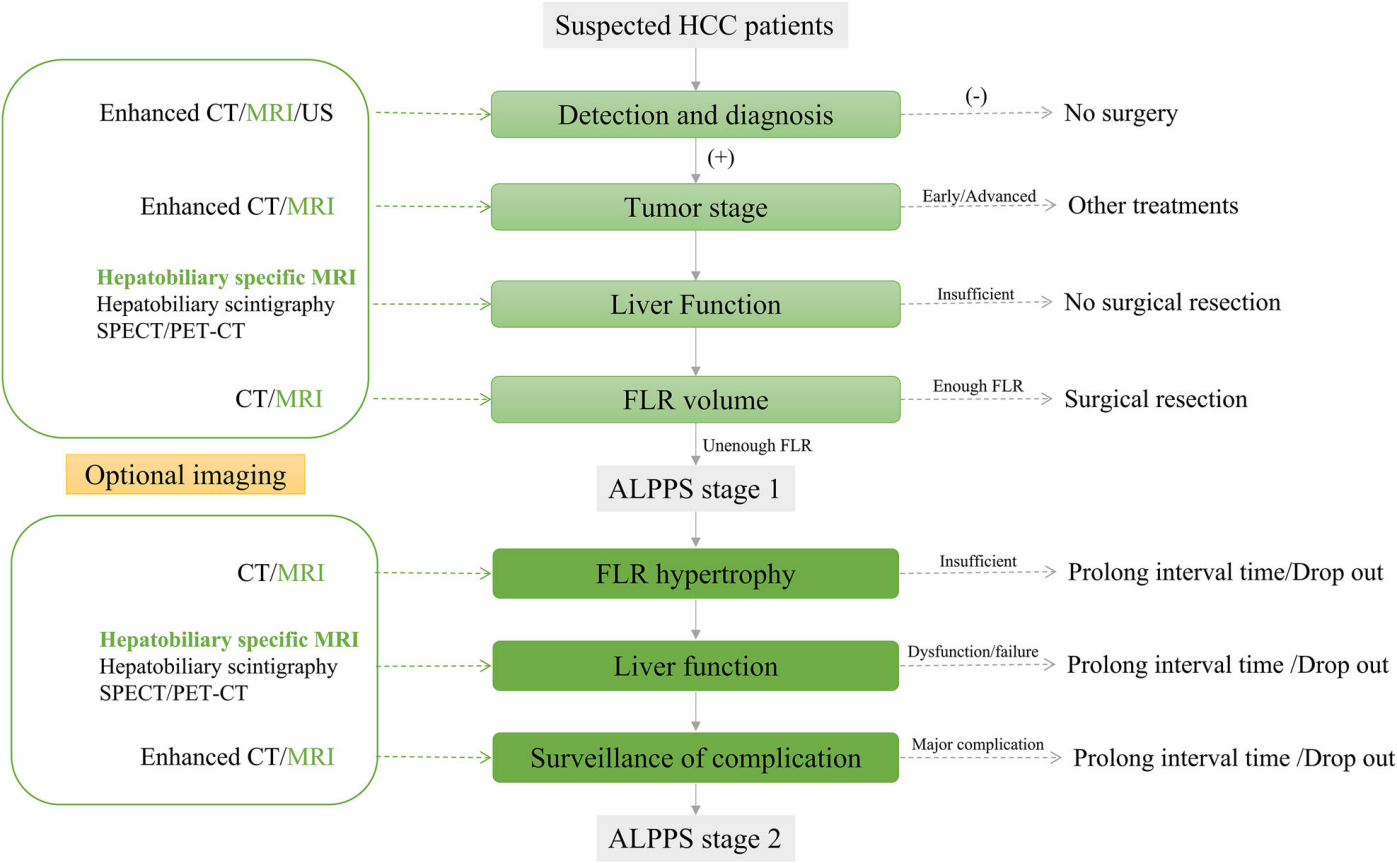
Medical imaging of patient selection and surveillance for ALPPS

In summary, ALPPS represents a powerful strategy to expand resectability in patients with initially unresectable liver tumors, but its success hinges on meticulous imaging-guided management. This review underscores the critical role of multimodal imaging (Fig. 11)—particularly hepatobiliary-specific MRI—in enabling safe patient selection, monitoring remnant liver regeneration, and detecting complications. With increasing evidence supporting its utility (Table 2), optimized imaging protocols offer a pathway to maximize benefits and mitigate risks throughout the ALPPS process.

**Table 2:** Key imaging assessments in ALPPS pathway: clinical indications and supporting evidence

Phase	Parameter	Recommended modality	Rationale/evidence summary	LoE	SoR [17]
Pre-op	Tumor staging and anatomical variation	Contrast-enhanced CT/MRI	High spatial resolution; essential for surgical planning	M	Strong
Pre-op	FLR volume	Volumetric CT or MRI	Widely used; reproducible measurements	M	Strong
Pre-op	Regional liver function	Gadoxetate MRI or 99mTc-GSA SPECT/CT	Detects functional deficits despite adequate volume	M	Strong
Interstage	FLR growth rate	Serial MRI/CT with volumetry	Weekly monitoring until threshold reached	L	Conditional
Interstage	Functional maturation of FLR	Gadoxetate MRI (hepatobiliary phase)	Identifies immature regeneration despite volume gain	M	Strong
Interstage	Hemodynamic complications	Doppler US/CT angiography	Detects portal vein recanalization or arterial steal	L	Conditional
Post-op	Complication screening	Contrast-enhanced CT/MRI	Gold standard for fluid collections, bleeding, thrombosis	M	Strong
Follow-up	Recurrence surveillance	Dynamic MRI every 3–6 mo	Higher sensitivity for small HCC recurrences	Low	Conditional

Strength of recommendation: Strong: The modality is recommended for most patients undergoing ALPPS or extended hepatectomy due to the favorable balance of benefits, accuracy, and safety. Conditional: The choice depends on institutional expertise, availability, and patient-specific factors

*LoE* level of evidence, *SoR* strength of recommendation

## Data Availability

The datasets generated and analyzed during the current study are not publicly available because they contain sensitive patient information but are available from the corresponding author upon reasonable request.

## Abbreviations

ALPPS: Associating liver partition and portal vein ligation for staged hepatectomy
BSA: Body surface area
CRLM: Colorectal cancer with liver metastasis
CT: Computed tomography
FLF: Future liver remnant function
FLR: Future liver remnant
FLV: Future liver remnant volume
HCC: Hepatocellular carcinoma
MRI: Magnetic resonance imaging
PHLF: Post-hepatectomy liver failure
PVE: Portal vein embolization
PVL: Portal vein ligation
TACE: Transarterial chemoembolization
